# Lifetime Prevalence of Sexual Violence and Its Associated Factors among High School Female Students in Jarso District, Oromia Region, Eastern Ethiopia

**DOI:** 10.1155/2021/1821579

**Published:** 2021-12-27

**Authors:** Fufa Dufera, Jemal Yusuf Kebira, Tesfaye Gobena, Nega Assefa

**Affiliations:** ^1^Jarso District Health Office, Oromia Regional Health Bureau, Minister of Health, Ethiopia; ^2^School of Public Health, College of Health and Medical Sciences, Haramaya University, Harar, Ethiopia; ^3^Department of Environmental Health, College of Health and Medical Sciences, Haramaya University, Harar, Ethiopia; ^4^School of Nursing and Midwifery, College of Health and Medical Sciences, Haramaya University, Harar, Ethiopia

## Abstract

**Background:**

Sexual violence is a serious public health problem affecting millions of young girls and women across the world. Recently, the issue of sexual violence against schoolgirls has garnered global and national attention with implications for health and education outcomes. Sexual violence is driven by a multitude of risk factors that occur at different levels. Understanding the magnitude, risk factors, and conceptuality of sexual violence is crucial for setting priorities and elimination efforts at different levels. Therefore, the objective of this study was to determine the lifetime prevalence of sexual violence and associated factors among high school female students in Jarso district, Oromia region, eastern Ethiopia.

**Methods:**

A school-based cross-sectional study was conducted in public high schools of Jarso district, eastern Ethiopia, from 1^st^ March to 5^th^ April 2019. A multistage sampling technique was used to select 559 eligible study participants. Data were collected by a structured self-administered questionnaire. The outcome measure of interest was lifetime sexual violence. Bivariate and multivariable logistic regression analyses were done. Statistically significant association of variables had been declared based on the adjusted odds ratio (AOR) with its 95% CI and *p* value < 0.05.

**Results:**

The overall magnitude of sexual violence among female students was 28.6% (95% CI: 25%-32.2%) in the study area. Forty (7.2%) participants have experienced coercive sex against their consent. Participant's level of education ((AOR = 1.5, 95% CI (1.03–2.30)), being unmarried ((AOR = 2.80, 95% CI (1.40–5.81)), consumption of alcohol ((AOR = 3.41, 95% CI (1.11–10.40)), using substances (hashish and/or shisha) ((AOR = 2.6, 95% CI (1.02–6.50)), and ever initiated sexual intercourse ((AOR = 5.9, 95% CI (3.3–10.7)) were positively and statistically associated with sexual violence at *p* value < 0.05.

**Conclusion:**

The overall magnitude of sexual violence was relatively high (28.6%). Thus, any intervention aimed to address sexual violence should consider the identified associated risk factors in the study area.

## 1. Introduction

Sexual violence (SV) is one of the most frequent forms of gender violence in which a person's sexual freedom is violated. It is a major public health problem affecting millions of girls and young women across the world [1]. According to the World Health Organization (WHO) estimates, 1 in 3 women aged 15-45 years worldwide experienced some form of sexual violence in their lifetime [2]. More specifically, more than 150 million young girls experienced some form of sexual violence below 18 years of age [3]. Despite the global epidemic, the prevalence of sexual violence against women is alarmingly high in developing countries, particularly in sub-Saharan Africa [4].

Approximately, one in 10 adolescents worldwide reported experiencing sexual violence [5]. Sexual violence against adolescents occurs in different circumstances, forms, and settings [6]. Adolescent schoolgirls are at risk of different forms of sexual violence by different perpetrators. Male teachers, school administrators, and students are common perpetrators of sexual violence against schoolgirls [7, 8]. They also experienced sexual violence at home or on the journey to and from school [9, 10].

Sexual violence has been found to have devastating effects on victims' health: physical, reproductive, psychological, and well-being [11–14]. Studies revealed that sexual violence against schoolgirl has negative effects on the girls' educational attainment [15–17]. Moreover, sexual violence against young girls also has a negative impact on the country's human and economic development [18–21].

Recently, the issue of sexual violence against schoolgirls has garnered global attention with implications for health and education outcomes [22–24]. With sexual violence becoming widespread in schools, the government of Ethiopia has enacted various policies and codes of conduct aimed at the prevention and control of school-related sexual violence [25–27]. In addition, the government of Ethiopia included ending violence against women in higher education as a priority concern in the second growth and transformation plan (2015-20) [28]. Furthermore, gender-responsive pedagogy addressing school-related gender-based violence through the curriculum has been implemented since 2014 [29]. Despite all these efforts, sexual violence against schoolgirls has remained the major challenge in the country's education systems, especially in high schools, with the recent studies on sexual violence among high school female students reporting that 74% and 41.6% of high school female students in central and Southwest Ethiopia, respectively, had experienced sexual violence [30, 31].

Sexual violence is driven by a multitude of risk factors that occur at the level of individuals, relationships, family, community, and the broader society. Consequently, understanding the magnitude, risk factors, and conceptuality of sexual violence is essential for setting priorities and elimination efforts at different levels [3, 32]. Taking into account the prior scenario and given that most studies conducted in eastern Ethiopia have been limited to urban areas [33, 34], with no consideration of young schoolgirls in rural areas, this study is aimed at determining the lifetime prevalence of sexual violence and associated factors among high school female students of Jarso district, Oromia region, eastern Ethiopia. The findings of this study may have a vital role in influencing policymakers and different stakeholders working on sexual violence prevention in the study area.

## 2. Methods

### 2.1. Study Area and Period

The study was conducted in Jarso district, East Hararghe Zone, Oromia regional state, eastern Ethiopia, from 1^st^ March to 5^th^ April 2019. The Jarso District is located in eastern Oromia, 561 km far from Addis Ababa, the capital city of Ethiopia, and 36 km from the Zonal capital, Harar town. It is bounded by Kombolcha district in the West, Dire Dawa Administration in the North, Gursum District in the South, and Chinaksen District in the East. The total population (2019 projection based on the 2007 Census, CSA) of the district was 321,868, of which 163,128 were males and 158,740 were females. The district has 52 schools of which 48 are primary schools and 4 secondary schools. The educational service coverage of the district was 100% (personal communication with the head of Jarso district educational office, 2019).

### 2.2. Study Design, Population, and Variables

An institutional-based cross-sectional study design was employed for this study. The study's population encompassed all female students attending secondary education in selected high schools of Jarso district. Thus, a total of 559 female students who met the eligibility criteria (attending a regular program in public schools) were enrolled in the study. Female students who were absent and seriously ill during the data collection period were excluded. The measurable outcome variable under study was lifetime sexual violence against female students.

### 2.3. Sample Size and Sampling Procedure

The sample size for this study was calculated by a single population proportion formula [*n* = (*Z* *α*/2)^2^ *P* (1 − *P*)/*d*^2^] by considering assumptions: 13.2% prevalence of sexual violence among high school female students taken from a previous similar study conducted in Dilla town, Ethiopia [35], with 95% confidence level, 4% tolerable margin of error, design effect of 2, and 5% expected nonresponse rate. Thus, [*n* = 2(1.96)^2^ × 0.132(1 − 0.132)/(0.04)^2^] = 550; considering 5% expected nonresponse rate, which is 28, the final sample size of the study became 578.

A multistage sampling technique was used in sampling the study participants. First, three high schools were randomly drawn from the total of four high schools in the district. Next, to achieve representativeness, the study subjects in each randomly selected high school were stratified based on their schooling level (grade 9 and grade 10). Then, the total female students in each stratum of respective high schools were identified and their sampling frame prepared. Then, proportional allocation to sample size was undertaken to determine the number of participants from each stratum (grade level) of the respective high school. Finally, study participants were selected through a simple random sampling technique using their classroom identification number as a sampling frame. Thus, the study recruited a total of 578 (328 from grade 9 and 250 from grade10) female students.

### 2.4. Data Collection Tools and Quality Control

Data were collected using a pretested and self-administered structured questionnaire. The questionnaire was adapted from gender-based violence tool assessment [36] and global early adolescent study tool [37] and validated by the WHO and another similar study [35]. The questionnaire consisted of the sociodemographic characteristics, family-related factors, sexual history and substance use condition, and incidence of sexual violence and its characteristics. The questionnaire was initially prepared in English and then translated into the local language (Afan Oromo). The Afan Oromo version was again translated back to English to check for any inconsistencies. Six diploma nurses and two-degree holder public health officers, a total of eight personnel participated in the data collection and supervision processes, respectively. The training was given for all personnel on the study procedures, data collection steps, and supervision activities, mainly focused on the responsibilities of data collectors, supervisors, and rights of respondents for two days. The questionnaire was checked for consistency and appropriateness to the study participants by conducting a pretest on 10% of the sample size at Felana high school in Kombolcha district (the nearest nonstudied district similar to the study subjects) fifteen days ahead of the actual data collection. Strict follow-up and supervision had been undertaken throughout the data collection period daily.

### 2.5. Data Processing and Analysis

Collected data were coded, entered, and cleaned using Epidata version 3.1 and exported into SPSS version 20 for analysis. Both descriptive and analytical statistics were executed. Descriptive statistics like mean, frequency, and percentage were used to describe the characteristics of participants using tables and text. To determine the association between independent and outcome variables, bivariate and multivariable logistic regression analyses were carried out. Factors found to have a *p* value < 0.25 in the bivariate logistic regression analysis were retained and entered into multivariable logistic regression analysis. The crude odds ratio and adjusted odds r1atio (AOR) were calculated with 95% confidence intervals (CI). Finally, a statistical significance is considered at a *p* value < 0.05.

## 3. Results

### 3.1. Sociodemographic Characteristics of Study Participants

A total of 559 female students participated in the study, yielding a response rate of 96.7%. The mean age of the study participant was 15.5 (SD ± 2.02) years. More than half, 320 (57.2%), of the study participants were under 15 years. The majority of participants 470 (84.1%) and 533 (95.3%) were Muslim in religion and Oromo in ethnicity, respectively. About one-third, 371 (66.4%), of the participants were rural residents. The majority of participants 465 (83.2%) were never married. Among those who were ever married, 56 (56.8%) of the participants were currently in union with their partners. About three-fourths, 420 (75.1%), of the participants' parents were living together (Table 1).

### 3.2. Sexual History and Substance Use Conditions of the Study Participants

More than half, 326 (58.3%), of the participants have regular boyfriends. One hundred ninety-two (34.3%) of the study participants have had initiated sexual intercourse with the mean age of first sexual intercourse 15.54 years. Of these, seventy-one (37%), thirty-seven (19.3%), and twenty-seven (14%) participants ever had started sexual intercourse due to marriage, personal desire, and peer pressure, respectively. Among the study participants who had initiated sexual intercourse, 32 (16.7%) of them have reported use of condom during their first sexual intercourse and more than half, 102 (53.1%), of them have had initiated first sexual intercourse at the age of under 15 years. With respect to substance use, the majority, 527 (94.3%), of the study participants did not consume alcohol while 195 (34.9%) of them have reported using Khat (Table 2).

### 3.3. Prevalence and Characteristics of Sexual Violence among the Study Participants

A little over a quarter, 160 (28.6%) (95% CI: 25%-32.2%), of the study participants have experienced some form of sexual violence at a point in their lifetime. The most common form of sexual violence experienced by participants was uninvited kiss 127 (22.7%) followed by use of verbal abuse 115 (20.6%). Another frequent form of sexual violence experienced by the participants involved unwelcome touching of their private parts 100 (17.9%). In addition, 76 (13.6%) and 40 (7.2%) of the study participants have experienced, attempted, and completed coercive sex against their consent in their life, respectively. Of the total completed coercive sex cases, 17 (42.5%) were performed at the victims' home, 15 (37.5%) at the perpetrator's home, and 8 (20%) at other sites including in schools, hotels, and forests. Boyfriends were the most claimed perpetrators of completed coercive sex cases (37.5%), followed by relatives (30%). Moreover, 16 (40%) of the study participants experienced coercive sex two and above times (Table 3).

### 3.4. Factors Associated with Sexual Violence among the Study Participants

Initially, the sociodemographic characteristics of respondents, sexual history, and risk behavior, as well as family-related factors, were analyzed with bivariate logistic regression to determine factors associated with sexual violence. Next, variables that were associated with sexual violence at a *p* value < 0.25 in the binary logistic regression analysis were fitted into a multivariate logistic regression analysis. In the final analysis, the respondent's level of education, marital status, alcohol consumption, substance use (hashish and/or shisha), and ever engagement in sexual intercourse were positively and statistically associated with sexual violence at a *p* value less than 0.05.

Female students who are attending grade ten were 1.5 times more likely (AOR = 1.5, 95% CI (1.03–2.30)) to experience sexual violence than those attending grade nine. Unmarried female students were three times more likely to face sexual violence compared to their counterparts ((AOR = 2.80, 95% CI (1.40–5.81)). Respondents who had drunk alcohol were three half times more likely ((AOR = 3.41, 95% CI (1.11–10.40)) to experience sexual violence compared to those who had not to drink. Similarly, respondents who used substances (Hashish and/or Shisha) were 2.6 more likely ((AOR = 2.6, 95% CI (1.02–6.50)) to experience sexual violence than nonusers. Moreover, female students who ever had initiated sexual intercourse were six times more likely to experience sexual violence compared to their counterparts ((AOR = 5.9, 95% CI (3.3-10.7)) (Table 4).

## 4. Discussion

This study is aimed at determining the magnitude of sexual violence and associated factors among high school female students in Jarso district, eastern Ethiopia. The overall magnitude of sexual violence among the study participants was 28.6% (95% CI: 25%-32.2%) in the study setting. In the final model analysis, the respondent's level of education, marital status, alcohol consumption, substance use (hashish and/or shisha), and ever-initiated sexual intercourse were positively and statistically associated with sexual violence.

Findings of the current study showed that over a quarter (28.6%) of female students have experienced some form of sexual violence in their lifetime, which is comparable to the previous findings reported in Brazil (30.5%) and Jima, Ethiopia (32.4%) [38, 39]. On the other hand, the result of the current study is lower than the findings of the study conducted among in-school students in Nigeria, 41.9%, and South Africa, 37.9% [40, 41], but higher than the findings of other studies conducted in Nigeria, Kenya, and Brazil, which reported 12.1%, 7.1%, and 4% of sexual violence [42–44], respectively. This variation could be due to the differences in the measurement of sexual violence, socioeconomy, and interventions made across the countries. Higher patterns of violence against girls are common in settings with low gender equality and women empowerment [45, 46].

Moreover, the magnitude of sexual violence in the current study is lower than the finding of previous studies done across different regions of Ethiopia: 74%, 68%, 41.5%, and 37.2% in Bishoftu and Mojo, eastern Ethiopia, Benchmaji, and Wolaita Sodo, respectively [30, 31, 33, 47]. In contrast, this study found a higher magnitude of sexual violence compared to the findings of studies conducted in Dilla town (13.2%), Debra Markos (24.2%), and Gurage Zone (15.9%) [35, 48, 49]. Thus, it is generally accepted that the findings of different studies regarding the prevalence of sexual violence lack consistency across Ethiopian regions. The explanation for the inconsistency may be attributable to the differences in social perception of gender norms and culture of disclosing the incidents of sexual violence in different ethnic groups or societies across the country's regions. The incidence of sexual violence is high in societies with the prevalence of traditional gender norms that dictate behaviors and expectations for boys and girls in different ways [50, 51]. Furthermore, the incidents of sexual violence are low in societies with a culture of choosing silence in reporting sexual violence.

The current study showed an association between sociodemographic factors and sexual violence. Unmarried female students were three times more likely to experience sexual violence than their counterparts, which is similar to the findings of the study conducted in Ethiopia and China. The study conducted in Ambo high school found that unmarried female students were three times more likely to experience sexual violence than married school girls [52, 53]. This possibility may be due to sociocultural attitudes in society towards married women that enhance dignity and security as motherhood.

As found in this study, another sociodemographic factor significantly associated with sexual violence was the respondent's level of education. Being in grade 10^th^ increased the risk of experiencing sexual violence by 1.5 folds compared to being in grade 9^th^. This finding is consistent with the finding of a study conducted in Nekemte high school where being grade 10^th^ increased the risk of experiencing sexual violence by 2.8 folds compared to their counterparts [54]. But, contrary to the current finding, the study conducted in China and Uganda revealed that the likelihood of experiencing sexual violence decreases with increased grade attainment [23, 53]. This variation could be due to the differences in educating the schoolgirls about sexual issues according to their age and level of grade across countries.

The current study found that female students who used substances, either hashish or shisha, were 2.6 times more likely to be sexually violated compared to their counterparts. This finding is consistent with the finding of a study conducted in China among high school students, which reported that students using drugs like cocaine and shisha were two times more likely to be sexually violated compared to their counterparts [55]. The reason behind this association may be due to the fact that drugs can alter the takers' consciousness and motivate for violence. In addition, shisha is easily accessible in the study area nexus to Khat (local stimulant), and that the perpetrators who used these substances eventually commit sexual violence.

Ever consumption of alcohol was significantly associated with sexual violence in the present study. Female students who had a history of alcohol consumption were 3.41 times more likely to experience sexual violence compared to students who had not a history of alcohol consumption. This finding is in line with other studies conducted in Ethiopia and elsewhere. A study conducted in China, Brazil, and South Africa found that female students who consumed alcohol were at increased risk of experiencing sexual violence compared to their counterparts [41, 43, 55]. Likewise, a study conducted in Debra Markos, Dilla town, Hadiya Zone, and Nekemte town, Ethiopia, revealed that alcohol consumption was one of the risk factors contributing to sexual violence among school girls [35, 48, 54, 56]. The possible reason may be schools are alcohol-free so perpetrators take females to a secret place where easily accessible to alcohol which enables them to be susceptible to violence.

Furthermore, the current study found ever having had sexual intercourse was associated with sexual violence. Female students who ever have had sexual intercourse were six times more likely to experience sexual violence than those who had not had sexual intercourse and is in line with the study done in Dilla and Nekemte high school [35, 54], which reported that female students who ever had sexual intercourse were five and six times more likely to experience sexual violence than their counterparts, respectively. Sexual violence in this study includes sexual experiences that female students were forced to participate in, including rape, and this could explain the increased risk of sexual violence among those who had had sexual intercourse. In addition, the possible explanation for this association may be those who had previous sexual experience might be easily approachable and be susceptible to conditions of violence as many of them were victimized by their boyfriends.

## 5. Conclusion

The overall magnitude of sexual violence among the study participants was 28.6% (95% CI: 25%-32.20%), of which forty (7.2%) participants have experienced coercive sex against their consent in their life. Boyfriends were the major perpetrators of forced sex. The respondent's level of education, marital status, alcohol consumption, substance use (hashish and/or shisha), and ever engagement in sexual intercourse were significantly associated with sexual violence. Therefore, it will be crucial if any proposed intervention aimed to address sexual violence shall consider the factors that were identified in the study area.

### 5.1. Limitations of the Study

The study may be exposed to social desirability and recall bias because the study was conducted on very sensitive, private issues and lifetime experience. Being a cross-sectional study design, this study does not establish cause-effect relationships between the study variables. In addition, this study recruited a limited sample size, and hence, the finding of this study may not be generalizable to female students in Ethiopia. Furthermore, the study was not incorporated qualitative interpretation of sexual violence.

## Figures and Tables

**Table 1:** Sociodemographic characteristics of high school female students in Jarso district, Oromia region, eastern Ethiopia, 2019 (*n* = 559).

Variables	Categories	Frequency	Percentages
Age group	<15	320	57.2
	15-19	210	37.6
	>19	29	5.2

Religion	Muslim	470	84.1
	Non-Muslim	89	15.9

Ethnicity	Oromo	533	95.3
	Amhara	22	3.9
	Others^∗^	4	0.8

Residence	Urban	188	33.6
	Rural	371	66.4

Educational status	Grade 9	317	56.7
	Grade 10	242	43.3

Mother's educational level	No formal education	318	56.89
	Primary	103	18.43
	Secondary	96	17.2
	College and above	42	7.5

Father's educational level	No formal education	264	47.2
	Primary	128	22.9
	Secondary	105	18.8
	College and above	62	11.1

Have ever married	Yes	94	16.8
	No	465	83.2

Currently married (94)	Yes	56	59.6
	No	38	40.4

With whom you live currently	With parents	416	74.4
	With friend	39	7.0
	With husband	39	7.0
	With relatives	40	7.2
	Other^∗∗^	25	4.4

Current parental living conditions	Mother and father live together	420	75.1
	Separated	79	14.1
	Only father alive	21	3.8
	Only mother alive	29	5.2
	Both are not alive	10	1.8

Receive enough money	Yes	179	32.0
	No	380	68.0

Monthly average family income	<1000 ETB	267	47.8
	1000-2500 ETB	154	27.5
	>2500 ETB	138	24.7

Notes: ETB: Ethiopian birr; others^∗^: Somali, Harari, and Gurage; others^∗∗^: both relatives and parents, alone.

**Table 2:** Sexual history and substance use condition among high school female students in Jarso district, Oromia region, eastern Ethiopia, 2019 (*n* = 559).

Characteristics	Categories	Frequency	Percentages
Have regular boyfriend	Yes	326	58.3
	No	233	41.7

Have you ever had sexual intercourse	Yes	192	34.3
	No	367	65.7

Age at first sexual intercourse	<15	102	53.1
	15-17	62	32.3
	≥18	28	14.6

Number of sexual partners (192)	One	56	29.2
	Two	88	45.8
	Three and above	48	25.0

Used condom during first sexual intercourse (192)	Yes	32	5.7
	No	527	94.3

Have ever drink alcohol	Yes	32	20.6
	No	444	79.4

Frequency of alcohol drinking	Daily	11	34.4
	1-2/week	3	9.4
	2-3/month	12	37.5
	Once/month	6	18.7

Have ever chewed Khat	Yes	195	34.9
	No	364	65.1

Frequency of chewing Khat	Daily	65	33.3
	1-2/week	48	24.6
	2-3/month	36	18.5
	Once/month	46	23.6

Have ever used substance like hashish and/or shisha	Yes	38	6.8
	No	521	93.2

**Table 3:** Characteristics of sexual violence among high school female students in Jarso district, Oromia region, eastern Ethiopia, 2019 (*n* = 559).

Characteristics	Categories	Frequency	Percentages
Experienced verbal abuse that undermines one's self-esteem	Yes	115	20.6
	No	444	79.4

Experienced uninvited kiss	Yes	127	22.7
	No	432	77.3

Experienced unwelcoming touch like breast, genitalia area	Yes	100	17.9
	No	459	82.1

Experienced forced sexual attempt without your consent	Yes	76	13.6
	No	483	86.4

Experienced coercive sex without your consent	Yes	40	7.2
	No	519	92.8

Place where coercive sex encountered	Victims' home	17	42.5
	Perpetrators' home	15	37.5
	Others^∗^	8	20.0

Perpetrators of coercive sex	Boyfriend	15	37.5
	Husband	6	15.0
	Relative	12	30.0
	Others^∗∗^	7	17.5

Age of the perpetrators	Equal to victims	15	37.5
	Older than victim	21	52.5
	Younger than victim	4	10.0

Frequency of encountered forced sex in your life	Once only	24	60.0
	Two times and above	16	40.0

Notes: others^∗^ = in school, hotel, and forest; others^∗∗^ = teacher, student, neighbor, and stranger.

**Table 4:** Factors associated with sexual violence among high school female students in Jarso district, Oromia region, eastern Ethiopia, 2019 (*n* = 559).

Variables	Categories	Sexually violated?		COR (95% CI)	AOR (95% CI)
		Yes	No		
Age	<15	103	217	1.55 (1.04-2.33)^∗^	1.15 (0.74,1.8)
	15-19	46	164	2.44 (.98-3.11)	1.23 (0.44-2.58)
	>19	11	18	Ref.	Ref.

Educational status	Grade 10	84	158	1.69 (1.2-2.4)^∗^	1.5 (1.03-2.3)^∗∗^
	Grade 9	76	241	Ref.	Ref.

Mother's educational level	No formal education	117	201	3.01 (1.05-6.1)^∗^	1.60 (0.66-3.90)
	Primary	25	78	1.7 (0.74-3.80)	1.01 (0.45-2.23)
	Secondary	18	78	0.88 (0.67-1.2)	1.2 (0.51-2.80)
	College and above	10	32	Ref.	Ref.

Father's educational level	No formal education	94	170	1.35 (1.02-2.4)^∗^	0.29 (0.06-1.45)
	Primary	32	96	1.37 (0.99-1.9)	0.43 (0.9-1.91)
	Secondary	25	80	1.35 (0.95-1.9)	0.37 (0.08-1.76)
	College and above	9	53	Ref.	Ref.

Currently living condition	With mother and father	95	322	Ref.	Ref.
	With friends	20	19	3.6 (1.85-7.04)^∗^	1.5 (0.57-3.92)
	With husband	15	24	2.1 (1.1-4.20)^∗^	0.83 (0.21-2.15)
	With relatives	18	22	0.88 (0.71-1.12)	2.1 (.97- 3.8)
	Others^∗∗^	13	12	3.7 (1.6-8.4)^∗^	0.25 (0.23-1.22)

Current parental living condition	Live together	100	320	Ref.	Ref.
	Separated	33	46	2.3 (1.4-3.80)^∗^	1.14 (.59-3.61)
	Only father alive	7	14	1.6 (0.63-4.07)	2.04 (0.75-5.46)
	Only mother alive	16	13	3.94 (1.83-8.5)^∗^	2.1 (0.81-6.01)
	Both are not alive	4	6	2.13 (0.58-7.7)	2.45 (0.7-9.4)

Marital status	Married	36	58	Ref.	Ref.
	Unmarried	124	341	0.58 (0.39, 0.93)^∗^	2.80 (1.4-5.80)^∗∗^

Ever drink alcohol	Yes	23	9	7.28 (3.31-16.1)^∗^	3.41 (1.1-10.4)^∗∗^
	No	137	390	Ref.	Ref.

Ever chew Khat	Yes	91	104	3.75 (2.51-5.45)^∗^	1.45 (0.9-2.32)
	No	69	295	Ref.	Ref.

Ever use substances like shisha or hashish	Yes	27	11	7.21 (3.45-14.80)^∗^	2.6 (1.02-6.50)^∗∗^
	No	133	388	Ref.	Ref.

Had ever started sexual intercourse	Yes	106	86	7.14 (4.76-10.7)^∗^	5.9 (3.3-10.7)^∗∗^
	No	54	313	Ref.	Ref.

Monthly average family income	<1000 ETB	60	207	1.7 (1.05-2.8)^∗^	1.22 (0.75-1.98)
	1000-2500 ETB	81	73	1.14 (0.74-1.74)	0.70 (0.4-1.22)
	>2500 ETB	19	119	Ref.	Ref.

Notes: ^∗^significant at *p* value < 0.05 for COR; ^∗∗^significant at *p* value < 0.05 for AOR; ETB: Ethiopian birr; CI: confidence interval; COR: crude odd ratio; AOR: adjusted odd ratio; others^∗∗^ = both relatives and parents, alone.

## Data Availability

Data is available upon request from the corresponding author.

## Abbreviations

AOR: Adjusted odds ratio
CI: Confidence interval
COR: Crude odds ratio
ETB: Ethiopian birr
SD: Standard deviation
SPSS: Statistical Package for Social Science
km: Kilometer.

## Supplementary Materials

Supplementary Materials Questionnare.
