# Supplementary material for: Lifetime Prevalence of Sexual Violence and Its Associated Factors among High School Female Students in Jarso District, Oromia Region, Eastern Ethiopia
Source: Int J Reprod Med. 2021 Dec 27;2021:1821579. doi: 10.1155/2021/1821579 (PMC8723865; doi:10.1155/2021/1821579)
Supplement: Supplementary Materials — Questionnare. [file 1821579.f1.docx]

ANNEXES

Annex I: Participant Information sheet and consent form Students.

Introduction: You are invited to take part in a study. Before you decide whether to participate, please allow me to explain why the research is being done and what it would involve. Please take the time to read or to listen as I read the following information. Please stop me at any time to ask questions if there is anything that is not clear, or if you would like more information. You will be asked if you wish to participate in the study or not. If yes, you will be asked to sign this Informed consent form. Please keep the information you hear in the group confidential. This study is being conducted under the leadership of investigator Fufa Dufera. The study was approved by Institutional Health Research Ethics Review Committee, Haramaya University.

Title of Study: Lifetime prevalence of Sexual violence and its associated factors among high school female students in Jarso district, Oromia region, Eastern Ethiopia 2019.

Objectives of the study: the objective of this study is to assess the magnitude of sexual violence and associated factors among high school female students in the Jarso district. The purpose is to provide baseline data on the issue for a better understanding of the outcome on the side of the victim so that appropriate protection and services can be provided by the concerned body.

Procedure and duration: If you agree to take part in the study, we will ask you to sign this form. You will be asked also to respond to the following questions. This will take about an hour of your time.

Potential Risks and Benefits: There is no possible risk associated with participating in this study except, the time you spent responding to the questionnaire. You may find one or more questions that we ask to be discomforting, emotionally sensitive, or upsetting. You do not have to respond to any question that makes you uncomfortable. There would not be any direct payment for participating in this study. But the findings from this research may generate important information for governmental and non -governmental agencies that want to work on the violence prevention and control area and sets a baseline for future planning and policy design.

Confidentiality: The information that is collected during the interview will be kept private. No one will be told that you have participated in the study. The information that is collected will be used only for the purpose intended. The study team will make every effort to protect your privacy and maintain the confidentiality of all the information that you provide. Your name or other identifiers will not be included in any published documents from this study. Raw and processed data will be stored in a computer dedicated to this study that only the study team can access.

Voluntary participation/withdrawal: Your participation in this study is voluntary. If you decide not to participate, you will not lose any existing benefits to which you are entitled. If you decide to take part, you are free to skip any questions and withdraw at any time.

Who to contact for clarification if there are any questions or enquires at any time about the study or the procedures, please contact

Principal Investigator: Fufa Dufera

E-mail: fufadufera8@gmail.com

Mobile phone: +251-911263936

Haramaya University College of Health and Medical Sciences Institutional Research Ethical

Review Committee: Office phone: 0254662011, P.O.Box: 235, Harar.

Declaration of Informed consent: I have read/ was read to me/ the participant information sheet. I have clearly understood the purpose of the research, the procedures, the risks and benefits, issues of confidentiality, the rights of participating, and the contact address for any queries will be informed that have the right to withdraw from the study at any time or not to answer any question that I do not want. Therefore,

I declare my voluntary consent to participate in this study with my signature as indicated below.

Signature of participants________________________ Date_______________

Investigator statement and signature

I certify that the participant has been given ample time to read and learn about the study.

All questions and clarifications raised by the participant have been addressed.

Signature _______________

Annex II: Data Collection Tool English Version

The following question was prepared to assess the magnitude of sexual violence and associated factors among high school female students in the Jarso district. You are kindly requested to provide your answers by responding to this brief questionnaire, which should take no more than 30 minutes. Your name and specific identifying information are not required and confidentiality is guaranteed. Thank you for your valuable time.

School code__________________ Questionnaire code__________________

Name of Data Collector: ____________________ Signature___________ Date: _______

Name of Supervisor: _________________________ Signature________ Date_______

Instructions

1. Please do not put your name on the questionnaire since all responses are confidential.

2. This questionnaire should be filled-in by female students in high schools.

3. Follow the instructions for each question.

4. Return the questionnaire in the envelope provided.

Part –I Socio-demographic characteristics of the participants

| No | Questions | Response | Remark |
| --- | --- | --- | --- |
| 101 | How old are you? | ________years old. |  |
| 102 | What is your religion? | 1=Muslim 2=None-Muslim |  |
| 103 | What is your ethnicity? | 1=Oromo 2=Amhara  3=Other_______ |  |
| 104 | What is your educational level? | 1=Grade 9 2=Grade 10 |  |
| 105 | Where is your current residence? | 1=Urban 2= Rural |  |
| 106 | With whom do you live currently? | 1= with parents 2= with friend  3= with husband 4=with relatives  5= with others____ |  |
| 107 | Have you ever married? | 1=Yes 2= No |  |
| 108 | Are you in marriage currently? | 1=Yes 2= No |  |

Part –II Family history of the study participants

| 201 | What is the highest level of education your mother has completed? | 1=No formal education  2= Primary education  3=Secondary education  4= College and above |  |
| --- | --- | --- | --- |
| 202 | What is the highest level of education your mother has completed? | 1=No formal education  2= Primary education  3=Secondary education  4= College and above |  |
| 203 | What is your current parental living condition? | 1=Mother and Father live together  2=Separated  3= Only father alive  4=Only mother alive  5=Both are not alive |  |
| 204 | Generally how much is your family's monthly income in birr? | 1= <1000 2=1000-2500  3= >2500 |  |
| 205 | Do you think that you are receiving enough money according to your demand? | 1=Yes 2=No |  |

Part III: Sexual history and substance use condition of the study participants

| 301 | Do you have a regular boyfriend currently? | 1=Yes 2=No |  |
| --- | --- | --- | --- |
| 302 | If yes to q 301, why is it important for you to have a boyfriend? | 1=I want to be cared for  2=I want to be popular  3=I want to be loved for  4=I want to be protected  5= others, specify_______ |  |
| 303 | Have you ever had sexual intercourse? | 1=Yes 2=No |  |
|  | If yes for (q 303) how do you start sexual intercourse? | 1=In a marriage  2=Personal desire  3=Peer pressure  4 =For financial purpose  5= By forcing against you  6= others, specify_____ |  |
| 304 | What was your age at first sexual intercourse? | _____________years old |  |
| 305 | How many sexual partners or boyfriends have you experiences until now? | 1= One 2=two  3=Three and above |  |
| 306 | Did you/your partner use condom the first time you did it? | 1=Yes 2.=No |  |
| 307 | Have you ever drink alcohol? | 1= Yes 2=No |  |
| 308 | If yes to q (307), how often do you drink alcohol? | 1=Every day  2= 1-2/ week  3=2-3/ month  4= once a month |  |
| 309 | Have you ever chewing Khat? | 1= Yes 2= No |  |
| 310 | If yes to q (309), how often do you chew Khat? | 1=Every day  2= 1-2/ week  3=2-3/ month  4= once a month |  |
| 311 | Have you ever been used substances like Hashish and/or shisha? | 1=Yes 2= No |  |

Part IV: Sexual violence and its characteristics among the study participants

| 401 | Have you ever experienced verbal abuse that you found sexual and undermines one's self–esteem? | 1=Yes 2= No 3= I don’t remember |  |
| --- | --- | --- | --- |
| 402 | Has someone ever kissed you in a way that you did not want to be? | 1= Yes 2=No 3= I don’t remember |  |
| 403 | Has anyone ever touched you in your private parts like breasts, genitalia when you did not want them? | 1=Yes 2= No 3. I don’t remember |  |
| 404 | Has anyone ever attempted or forced you to have sexual intercourse when you didn't want to? | 1= Yes 2= No 3. I don’t remember |  |
| 405 | Did you ever have sexual intercourse when you didn’t want because you were afraid of what he might do? | 1= Yes 2= No |  |
| 406 | If Yes to q (405) where did you encountered forced sex? | 1= In your home  2= at his home  3= others place, specify_________ |  |
| 407 | If yes to q (405), who was the Perpetrator? | 1= Boyfriend  2=Husband  3=Relative  4=Others, specify___________ |  |
| 408 | If yes to q (305), how do you consider the age of the perpetrator /attacker? | 1= Equal to you  2= Older than you  3=Younger than you |  |
| 409 | If yes to q (305), how many times in your lifetime did you encountered such forced sex? | 1=once only  2= two times and above |  |
